# Identification of milk quality and adulteration by surface-enhanced infrared absorption spectroscopy coupled to artificial neural networks using citrate-capped silver nanoislands

**DOI:** 10.1007/s00604-022-05393-4

**Published:** 2022-07-29

**Authors:** Sherif M. Eid, Sherine el-Shamy, Mohamed A. Farag

**Affiliations:** 1 Analytical Chemistry Department, Faculty of Pharmacy, October 6 University, 6 October City, Giza, Egypt; 2 Pharmacognosy Department, Faculty of Pharmacy, Modern University for Technology & Information, Cairo, Egypt; 3 Pharmacognosy Department, Faculty of Pharmacy, Cairo University, Cairo, 11562 Egypt

**Keywords:** Surface-enhanced infrared absorption spectroscopy, Optical sensor, Artificial neural networking, Milk components, Citrate-capped silver nanoparticles, Localized surface plasmon resonance

## Abstract

Milk is one of the most important multicomponent superfoods owing to its rich macronutrient composition. It requires quality control at all the production stages from the farm to the finished products. A localized surface plasmon resonance optical sensor based on a citrate-capped silver nanoparticle (Cit-AgNP)–coated glass substrate was developed. The fabrication of such sensors involved a single-step synthesis of Cit-AgNPs followed by surface modification of glass slides to be coated with the nanoparticles. The scanning electron microscope micrographs demonstrated that the nanoparticles formed monolayer islands on glass slides. The developed surface-enhanced infrared absorption spectroscopy (SEIRA) sensor was coupled to artificial neural networking (ANN) for the qualitative differentiation between cow, camel, goat, buffalo, and infants’ formula powdered milk types. Moreover, it can be used for the quantitative determination of the main milk components such as fat, casein, urea, and lactose in each milk type. The qualitative results showed that the obtained FTIR spectra of cow and buffalo milk have high similarity, whereas camel milk resembled infant formula powdered milk. The most difference in FTIR characteristics was evidenced in the case of goat milk. The developed sensor adds several advantages over the traditional techniques of milk analysis using MilkoScan™ such as less generated waste, elimination of pre-treatment steps, minimal sample volume, low operation time, and on-site analysis.

**Graphical abstract:**

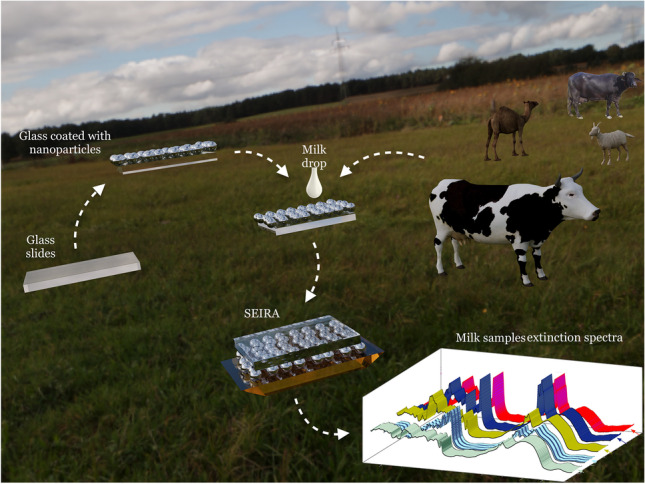

**Supplementary Information:**

The online version contains supplementary material available at 10.1007/s00604-022-05393-4.

## Introduction

Milk is the exclusive first food for every human. Milk presents a rich matrix of proteins, fats, vitamins, minerals, sugars, and enzymes, in addition to several bioactive factors that impart health benefits to humans [1]. The most widely used dairy product is cow milk, whereas goat milk has a lower incidence of allergic reactions and higher digestibility than cow milk [2]. Camel milk is one of the most important kinds of milk in Asia and Africa for decades owing to its benefits to human health including several health benefits, i.e., anti-carcinogenic properties, hypo-allergenic effect, and anti-diabetic properties, making it be used not only as dairy food but also as a remedy [3, 4].

Qualitative and quantitative determination of the different milk components and identification of its adulteration and impurities is an active area of research to ensure milk safety and quality [3, 5]. Multiple methods based on infrared spectroscopy (IR) have been developed for milk analysis targeting mostly analysis of milk fat, lactose, protein, and solid not fats [6]. In 1990 was the first use of Fourier transform infrared spectroscopy (FTIR) for milk analysis coupled with partial least squares (PLS) chemometrics regressions [7, 8]. Later, FTIR became the official method for milk analysis [9] providing quantitative and qualitative molecular infrared fingerprints of substances, based on their functional groups. FTIR is suited for on-site quality control in factories with its high efficacy and relatively low costs among other spectroscopic techniques, i.e., MS and NMR [8, 10–12]. Most of the methods are based on the PLS algorithm to expect the concentration, effect, or property of interest in the milk composition. More recently, algorithms other than PLS were employed to predict milk components and properties from milk FTIR spectra [13]. Other studies focused on the utilization of advanced localized surface plasmon resonance (LSPR) such as surface-enhanced Raman spectroscopy (SERS) for the rapid detection of foreign substances in the milk matrix [14–16].

Nanoparticle-based methods were widely used in multi-target sensing techniques in the last decade such as array-based sensing and SERS [17, 18]. Raman and infrared are the two faces of the vibrational spectroscopy coin, and they are complementary techniques. Soon after the discovery of the SERS effects, the new vibrational mode of surface-enhanced infrared spectroscopy (SEIRA) was unleashed, but SEIRA has received less attention than SERS due to its smaller enhancement factor but the sample preparation was easier. Despite vibrational spectroscopy by its nature having higher sensitivity than Raman spectroscopy, the sensitivity of SEIRA and that of SERS are comparable, and they can be used for the quantitative determination of picogram to microgram concentrations of molecules on the metal surfaces. They are complementary techniques; for example, SERS provides a completely different spectrum of p-aminothiophenol from the normal Raman spectrum. However, the SEIRA spectrum of the same analyte showed a slight change in its vibrational properties. The reason for the difference in SERS spectra is the photo-driven charge transfer mechanisms, and these conclusions were obtained with the help of SEIRA [19]. In another study, SERS and SEIRA showed the same sensitivity towards the same analytes on same plasmonic chips utilizing gold nanostars [20]. SEIRA attracted global interest through the development of the FTIR devices and nanoscience; many applications have been developed by controllable synthesis and characterization of nanoparticles and nanostructures. SEIRA has been applied most extensively to in situ and time-resolved studies of electrochemical interfaces. SEIRA has been applied most extensively to in situ and time-resolved studies of electrochemical interfaces and Solid surfaces such as human skin, glass, polymers, agricultural products, and semiconductors [21–23].

In comparison to traditional mid-IR spectrometry, SEIRA offers an alternative technique that has the same advantages as mid-IR techniques in addition to the utilization of the LSPR for IR signal enhancement [24]. LSPR is observed when the frequencies of incident photons match that of the collective oscillations of the conductive electrons of metal nanoparticles. Once the targeted chemicals in a matrix are exposed to the infrared light in close contact with the optimized metal islands, a LSPR is activated and the IR bands are enhanced by a factor of 2 or more orders of magnitude with improvement in both the accuracy and sensitivity level of analyte detection [25]. The intensity and frequency of the LSPR depend on the type of the nanoparticle material (silver, copper, gold, etc.), and its shape, size, and surrounding environment [26]. Different sensors based on LSPR have been applied in many important fields [27, 28].

In the current study, citrate-capped silver nanoparticles (Cit-AgNPs) were synthesized in a single-step reduction method. The fabricated Cit-AgNPs monolayers were further immobilized on the surface of modified glass slides to act as a substrate for SEIRA measurements. The developed SEIRA substrates are considered lab-on-chip sensors that can be employed for the qualitative and quantitative analyses of milk types. For qualitative purposes, each type of milk has its characteristic bands on the IR region which are considered a unique fingerprint for the milk types, so that any contamination or impurities in milk can be easily discovered as the impurities would lead to the appearance of abnormal bands on the IR spectrum of raw milk samples; i.e., the unique fingerprint of each milk will be altered. Moreover, the obtained characteristic IR spectra of cow, camel, goat, buffalo, and infants’ formula powdered milk can be used for identification and differentiation between milk types using visual IR spectrum inspection. For quantitative purposes, the developed SEIRA sensor coupled with an artificial neural networking (ANN) tool was applied for analysis of the main milk components such as fat, casein, urea, and lactose in each milk type to identify which milk further provides the best source of each macronutrient.

## Experimental section

### Materials and reagents

All the used reagents were of high-purity analytical grades; pure crystals of silver nitrate (AgNO_3_) (≥ 99.0%), trisodium citrate (TSC), 3-aminopropyltriethoxysilane (APTES, 99%), hydrogen peroxide (H_2_O_2_), sulfuric acid (H_2_SO_4_), hydrochloric acid (HCl), methanol, unflavored casein powder from milk, urea, and lactose monohydrate were obtained from Sigma-Aldrich, USA. The fat sample was purchased from New Zealand Milk Products (NZMP) Company, New Zealand. Milli-Q double-distilled purified water (Millipore Co., USA) was used in all assays. The milk sampling was performed on the farm level where farmers deliver the obtained milk from each animal (cows, buffalo, goats, and camels). Before the milk collection, the fresh milk was agitated in the container for 10 min on the farm. The samples were collected from the top of the tank using a sterilized dipper and transferred into sterilized test tubes. The samples were rapidly stored at − 20 °C until the analysis time. Each farmer delivers 1 L from each animal for 2 weeks. The infants’ powdered milk was obtained from Bebelac® formula, Danone Nutricia, Netherlands.

### Instruments

A FTIR (IRAffinity-1, Shimadzu, Japan) device attached to an attenuated total reflectance (ATR) unit type 8200HA (PIKE Tech, USA) was used for acquisition of the mid-infrared spectra. The FTIR device provides broadband (4600–400 cm^−1^) mid-infrared light using a ceramic high-energy light source, a germanium-coated KBr beam splitter, He–Ne laser light used for the optics alignment, and a temperature-controlled DLaTGS detector. The ATR unit includes a high-throughput trapezoid-shaped horizontal ZnSe prism with the following dimensions: 4 mm thick, 10 mm wide, and 80 mm long. The FTIR device is controlled by the IRsolution V.1.6 software (Shimadzu Co., Japan). MilkoScan™ milk analyzer (Model FT-1, Foss Electric, Hillerød, Denmark) used for comparative milk analysis. A double beam UV/VIS spectrophotometer model 1800-PC (Shimadzu Japan) controlled by the UVProbe V.2.4 software used for UV/VIS measurements. The photomicrographs of the nanoparticles were recorded using the JEOL transmission electron microscopy (TEM) device model JEM-1010 (Tokyo, Japan), while the photomicrographs of the substrate coated with nanoparticles were recorded using the scanning electron microscope (SEM) model JSM-6700F (Electro Co, Japan). A Malvern Zetasizer model Nano ZS 90 with multi-angle dynamic light scattering (DLS) equipped with a He–Ne laser (wavelength of 633 nm) and a backscatter detector at an angle of 173° was used for the measurement of nanoparticle size and its zeta potential. The device was controlled by the Malvern V.7.11 software. All ANN chemometric calculations were performed using MATLAB R2020b V.9.9 with Deep Learning Toolbox, from MathWorks, MA (www.mathworks.com).

### Citrate-capped silver nanoparticles

#### The preparation method

We have prepared the Cit-AgNP colloidal solution as described in [27, 28]. The details are in the supplementary file.

#### The characterization techniques

The prepared Cit-AgNP solution was centrifuged for 20 min at 6000 rpm. The precipitated nanoparticles were collected and redistributed in 350 mL of purified de-ionized water, and then a UV/Vis spectrum in the range of 600–200 nm was recorded.

The average size of Cit-AgNPs was measured using a Zetasizer device adjusted to general-purpose analysis. Each sample of the freshly prepared Cit-AgNPs was measured three times, and each measurement involved 15 runs for 10 s and then the measurements were averaged.

The fabricated Cit-AgNP size, shape, and dimensions were computed using the TEM micrographs. A drop of the Cit-AgNPs was placed over the surface of the carbon-coated copper grid. The drop was left to dry, and then the grid was scanned using the TEM instrument.

### Immobilization of citrate-capped silver nanoparticles on the glass substrate

The glass slides were dipped into a mixture of concentrated HCl and methanol (1:1 v/v) for 30 min to degrease the glass surface. The slides were rinsed several times using de-ionized water until the complete removal of HCl and methanol to prevent interaction with H_2_SO_4_ to be used in the following steps. The glass surface was cleaned by soaking into a hot piranha solution mixture of H_2_SO_4_:H_2_O_2_ (3:1 v/v) for 1 h to expose the (–O–) groups on the glass surface followed by rinsing with de-ionized water several times. The cleaned glass slides were immersed into an ethanolic solution of 1% APTES for 2 h for amino group functionalization via the salinization process. Thereafter, slides were ultrasonically cleaned in absolute ethanol to remove excess APTES, and placed in an oven at 120 °C for 1 to enhance the covalent binding.

Cit-AgNP thin films were prepared using wet chemical deposition of the capped nanoparticles on the surface of the salinized glass. The APTES-coated glass slides were dipped into a solution of citrate-capped silver nanoparticles for 12 h followed by air-drying. The dipped slides’ surface morphology has been examined using SEM.

### Qualitative and quantitative analysis of milk components

#### SEIRA measurement procedure

The ZnSe crystal has been installed on the FTIR device, and further purged using dry nitrogen gas aiming to prevent any interference of carbon dioxide gas or humidity during the measurements.

For the recording of a background spectrum, the glass coated with Cit-AgNPs was installed over the ZnSe crystal under moderate pressure applied using a pressure clip to guarantee full contact between the surface of the ZnSe crystal and the Cit-AgNPs attached to the surface of the glass slides.

For each measurement, 300 µL from each sample was injected over the surface of glass slides coated with the nanoparticles and they were left to dry. The slides were placed on the upper surface of the ZnSe horizontal crystal to be in direct contact with the samples, and a moderate pressure was applied.

Parameters for FTIR scanning were set as follows: scanning resolution 4, number of FTIR scans 60, mid-IR range 4600–400 cm^−1^, and the apodization function SqrTriangle. Each recorded FTIR spectrum yielded 2128 data points using 1.95 wave number intervals. The IR light penetrates the horizontal ZnSe prism with an angle of incidence equal to 45°; light is reflected 14 times inside the ZnSe crystal, producing evanescent waves that penetrate about 2 mm into the sample. The prism refractive index was found to be 2.43 with an effective path length that equals 12.13 mm. Between measurements, the prism surface was cleaned with water.

#### Preparation of the calibration and validation sets using milk components

An experimental set including 25 mixtures was prepared following a multilevel partial factorial experimental design, with each mixture measured in triplicate and scanned 60 times. The mixtures were prepared by appropriate dilution of their respective working solutions to yield calibration ranges corresponding to the reported values [3, 29, 30] of fat, casein, lactose, and urea. The concentration ranges were 15–75, 10–50, 15–75, and 0.1–0.5 mg/mL equivalent to 1.5–7.5%, 1–5%, 1.5–7.5%, and 100–500 mg/L for fat, casein, lactose, and urea, respectively. The experimental design presented in Suppl. Table 1S was prepared by using four factors (fat, casein, lactose, and urea) and five concentration levels for each milk component. Ten mixtures were prepared using the concentrations highlighted in Suppl. Table 1S and assigned as the validation set. The mixtures were scanned under the same conditions stated in “SEIRA measurement procedure.”

### ANN to identify milk components

The ANN modeling was developed using the prepared experimental set of five levels and four factors design as shown in Suppl. Table 1S. The experimental set was prepared by using four factors (fat, casein, lactose, and urea) and five concentration levels for each milk components following the partial factorial design as stated in “Preparation of the calibration and validation sets using milk components.” This partial factorial design spans the mixture space fairly well, where there are five mixtures for each compound at each concentration level, resulting in 25 mixtures. The recorded SEIRA spectra of the mixtures were used as the input layer and the 25 concentration levels as the output layer during the building of the ANN model. The input layer was composed of an array of size *P*_*ij*_ (2128 × 25) belonging to the set of SEIRA spectra of the mixtures, where its rows represent the absorbance for each of the 2128 wave numbers acquired in each spectrum.

**Fig. 1:**
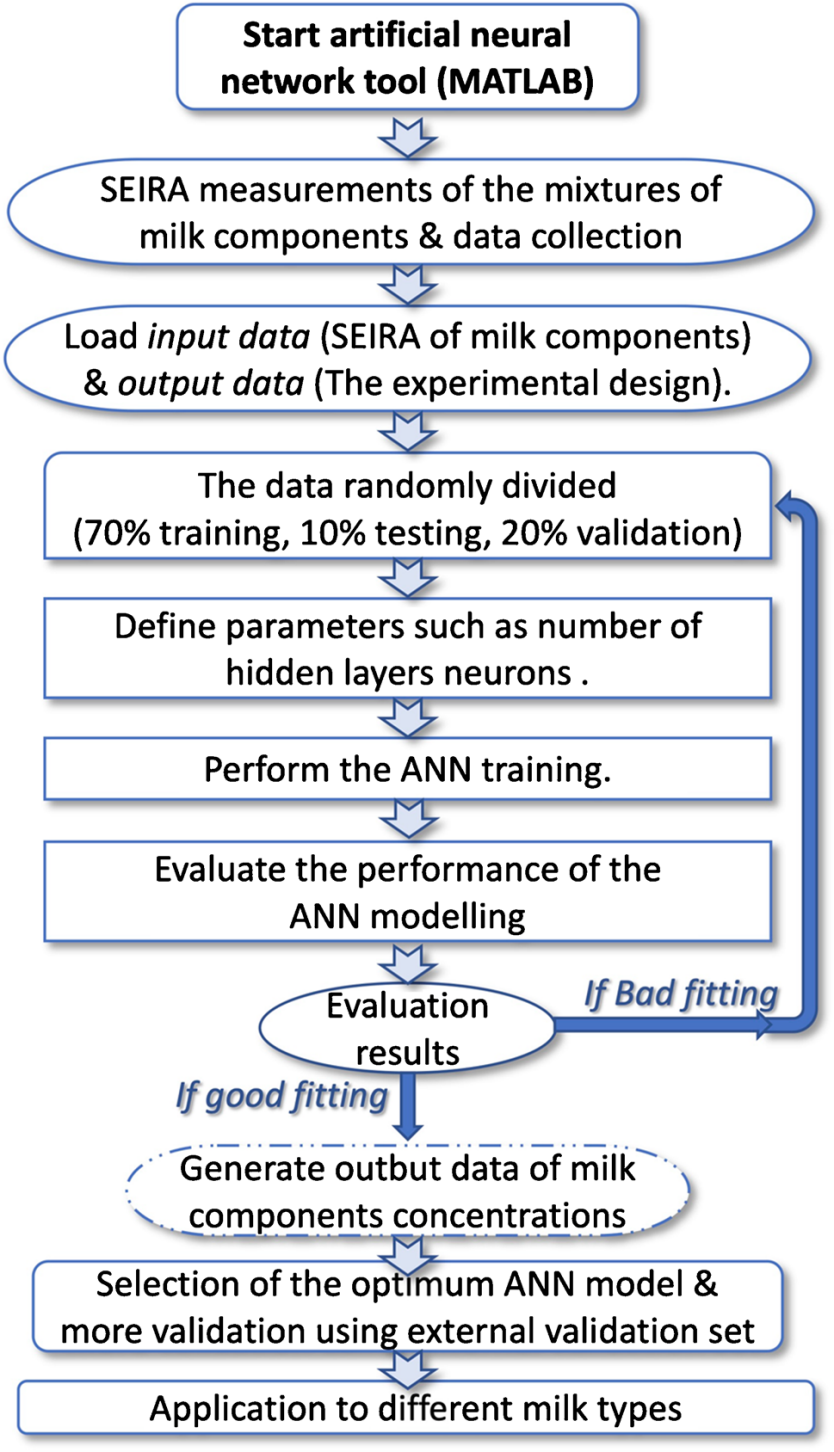
The framework diagram for the training of the developed ANN model

The architecture workflow employed (Supp. Figure 1S) consisted of an ANN with one hidden layer, using backpropagation learning with the Bayesian regularization algorithm for training the model as described in training diagram in Fig. 1. The performance index used was the mean square error (*MSE*) with the target criteria being less than or equal to 10^−10^ and error tolerance of ± 2% for the network output.

All the data were randomly presented to the artificial neural network model on the MATLAB software to distribute any possible noises and optimize for results. The network output was adjusted by a linear activation function to discrete intervals [1–4], chosen for convenience. Each of the four numbers refers to one of the milk components (fat, casein, lactose, and urea).

The ANN initial model was developed using 100% of the prepared experimental set mixtures, and so for further analysis, a subset of 70% of these mixtures was selected to create an ANN training set, leaving 20% for validation via cross-validation and 10% for the test set. The performance of the model was validated using an externally prepared validation set for testing the fully trained ANN model.

### Application to cow, buffalo, goat, camel, and commercial powdered infant’s milk samples

Fat, casein, lactose, and urea levels were determined in milk samples obtained from cow, buffalo, goat, camel, and commercial powdered infant’s milk. For the cow, buffalo, goat, and camel milk samples, exactly 300 µL from each sample was placed on the surface of the nanoparticle-coated glass substrate and measured as mentioned in “SEIRA measurement procedure.”

For infants’ powdered milk samples, the nutritional information on the marketed milk reports one spoonful (about 10 g) of the powder dissolved in 90 mL of water was enough for babies. One teaspoonful of the powder was thus dissolved in 100 mL water, with 300 µL from the prepared sample placed on the surface of the prepared glass substrate and measured as stated in “SEIRA measurement procedure.”

## Results and discussion

SEIRA bands are based on the chemical functional groups of analytes as each function group has its unique absorption band within the active mid-IR range (4600–400 cm^−1^); consequently, the SEIRA spectrum is considered a fingerprint for molecules [31]. These fingerprints can be used for the identification and differentiation of molecules [32]. In the current method, spherical or semispherical Cit-AgNPs were prepared by the reduction of AgNO_3_ using sodium citrate. Citrate salt acts as a capping and reducing agent of the prepared metal nanoparticles [33]. The prepared nanoparticles were attached to the silanized glass slides surface to yield a suitable SEIRA substrate which acts as a chemical enhancer for the quantitative analysis of the main milk components (i.e., fat, casein, lactose, and urea). The large surface area of Cit-AgNPs has a high capacity for adsorption of the under-investigation compounds on its surface. The LSPR interactions led to the enhancement of the IR absorption signals. This enhancement permitted the quantification of low molecule levels at high accuracy and robustness.

### Citrate-capped silver nanoparticle characterization

Cit-AgNPs were carefully prepared using the bottom-up technique that involved the reduction of AgNO_3_ to produce silver metal capped with the reducing agent [33]. The selection of the reducing agent is a key step as it affects the morphology, size, and charge on the surface of the nanoparticles. The weak reducing agent “trisodium citrate” was selected as it has dual functions; the first is the ability to work as a capping agent imparting a negative charge on the surface of nanoparticles. The second is the weak controllable reduction of AgNO_3_ to obtain the required size and shape [34]. Different methods were applied for the characterization of the prepared nanoparticles as discussed in the next subsections.

#### UV/Vis spectroscopy

Their spectrum is a rapid indicator of the shape and size of the Cit-AgNPs, with the wave number, shape, and width of the UV peaks differing according to the morphology of the nanoparticles. The Cit-AgNPs showed a narrow peak with a *λ*_max_ of 411 nm as shown in Suppl. Figure 2S, confirming the formation of spherically or mostly spherically shaped Cit-AgNPs of a size around 10–60 nm in accordance with the literature [35].

#### Dynamic light scattering calculation (Zetasizer)

Zetasizer was employed for measurement of the size of the nanosized particle at a *Z*-average of particle distribution by the intensity at 40 nm. size determined based on dynamic light scattering calculations involving triplet recording of the sample using the incident light angle of 90° at 25 °C. Suppl. Figure 3S illustrates the nanoparticle size distribution by the intensity which indicates that the most dominant *Z*-average of the particle size equals 40 nm; the smaller peaks indicate the presence of fewer smaller-sized nanoparticles. The obtained polydispersity index (*PDI*) was less than 0.7, indicating monodispersed nanoparticles.

#### Transmission electron microscopy micrographs

**Fig. 2:**
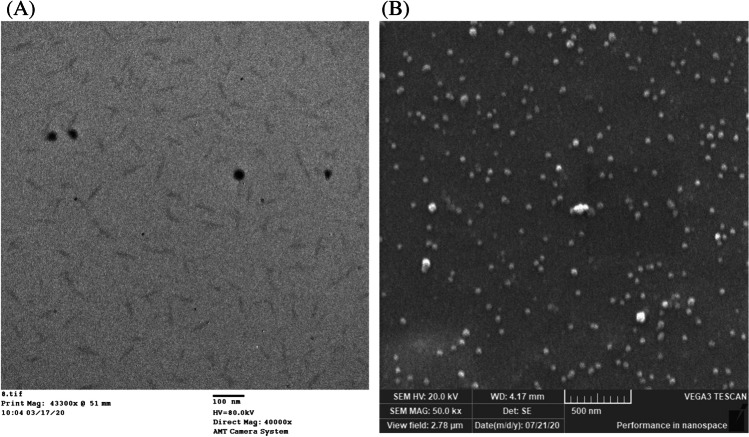
TEM micrograph of the prepared citrate-capped silver nanoparticles that appears spherical or semispherical in shape (**A**). SEM micrograph of the glass slides coated with citrate-capped silver nanoparticles showing islands of the nanoparticles attached to the surface of glass (**B**)

The prepared nanoparticles’ uniformity, distribution, shape, and size can be easily demonstrated using TEM as illustrated in Fig. 2, which shows spherically or semispherically shaped nanoparticles with no obvious aggregation. The average particle size was found to be 40 nm.

#### Nanoparticles’ long-term stability (UV/Vis and zeta potential)

Different techniques have been applied to confirm the stability of Cit-AgNPs for a long time. The first involved frequent recording of the UV/Vis spectrum of the prepared nanoparticles for 3 weeks, which revealed a slightly changed UV peak shape, with *λ*_max_ of 411 nm to indicate Cit-AgNP stability. The second involved the recording of nanoparticles’ zeta potential for 3 weeks targeting the monitoring of change in the surface negative charge of Cit-AgNPs. The calculated zeta potential equaling − 31 confirmed highly negatively charged Cit-AgNPs, consistent with the reported potential for stable negatively charged silver nanoparticles [36].

#### Surface examination using scanning electron microscopy micrographs

The localized surface plasmon resonance depends on the shape and thickness of the metal islands that were assembled over the surface of the salinized glass. The film’s thickness is crucial in the optimization of infrared absorption bands, as thick films may cause loss of spectral intensity. The salinized glass slides were dipped for 12 h into a solution of freshly prepared silver nanoparticles. The surface morphology was identified using TEM. Figure 2 shows a uniform thin layer formed with multiple metal islands attached to the surface of glass slides, with nanoparticles mostly uniform and spherical with a smooth surface. There were no irregular shapes related to any impurity that can be observed.

### Surface-enhanced infrared spectroscopy investigations

#### Qualitative and quantitative fingerprints of the milk components

**Fig. 3:**
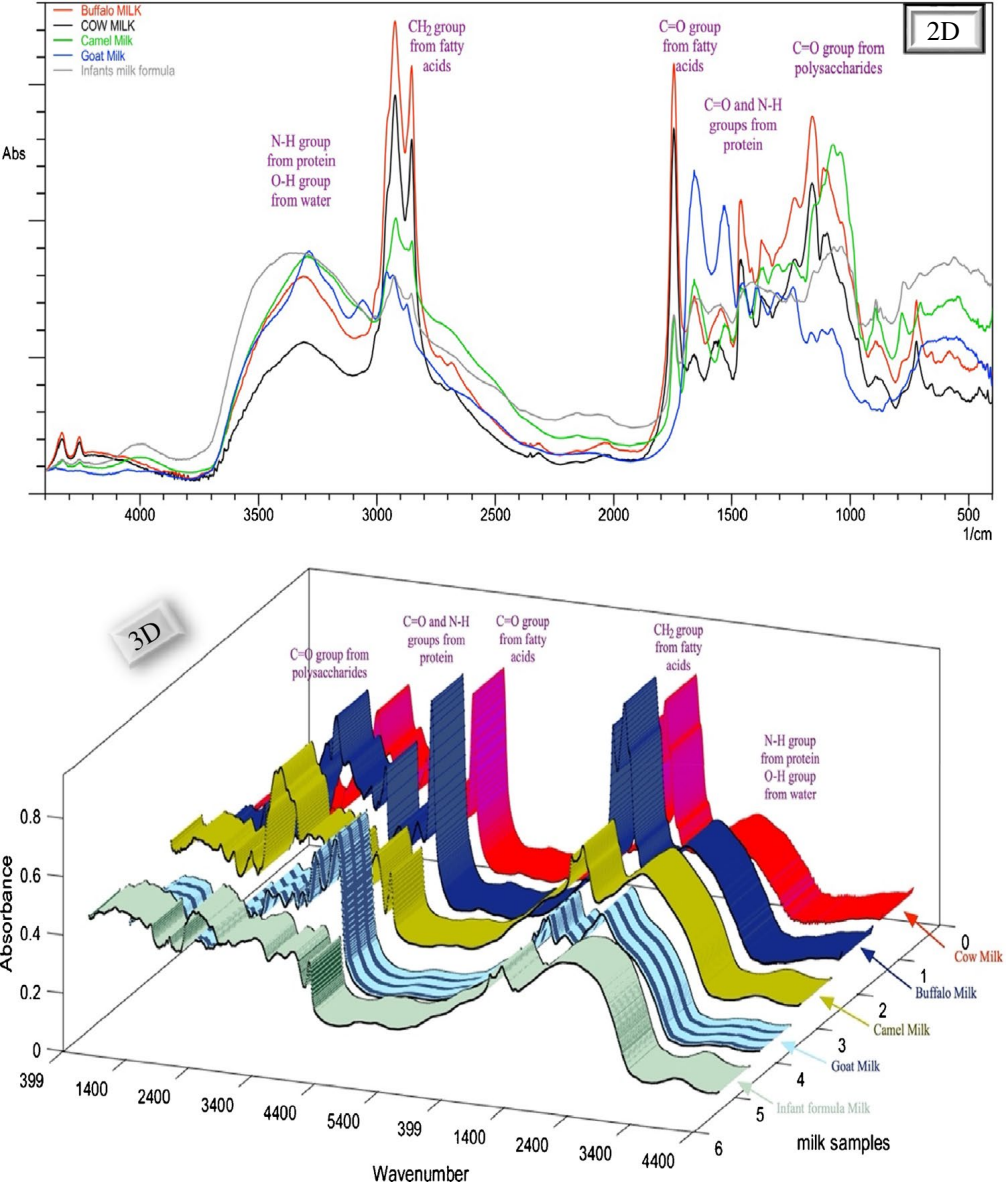
Fourier transform infrared spectroscopy (FTIR) spectra of cow, buffalo, camel, goat, and infant formula milk samples recorded and plotted as 2D and 3D overlay plots

The preliminary spectra of raw milk samples obtained from buffalo, cow, camel, goat, and infant powdered formula were recorded aiming for studying the structure–infrared bands’ relationship. Representative 2D and 3D spectra were obtained and are presented in Fig. 3. Each spectrum is considered a fingerprint for each milk type showing the characteristic bands that represent the structure of its main composition. All milk types share the main components such as fats and protein, but they differ in conjugation and concentration, so that the IR bands that represent the fat structure appear in the IR spectra of all milk types but with different intensities due to different fat concentrations of each milk. The differences in band intensity indicate different component levels as the absorption intensity of bands correlates with the concentration of the main milk components such as fat and casein. From the visual inspection, the FTIR spectrum of cow milk showed similarity to buffalo milk spectrum, whereas camel milk and infant formula milk spectra showed similarity. The goat milk spectrum showed many qualitative differences from other types appearing the most different. The higher band intensity at 2870 cm^−1^ in the buffalo milk spectrum corresponded to an acyl chain of fatty acids suggestive of higher fat levels in buffalo milk than those in cow milk, following that reported in the literature [37]. The degree of FTIR absorption bands of infant’s milk powdered formula corresponds to the nutritional information label on the commercial milk boxes.

Integration of FTIR peaks derived from the spectrum of each milk confirmed the structure of milk components in the mid-IR region. The characteristic functional groups of milk components showed typical bands with broad and strong (–OH) stretching between 3700 and 3000 cm^−1^ due to water content [38]. The sharp absorption bands of (–CH) mainly at 2870–1464 cm^−1^ are attributed to fat content [37]. The protein content was determined using the vibrational absorption bands of the amide I and amide II groups at nearly 1600–1548 cm^−1^ [39], along with an absorption stretching band of (O = P–O) at 1100 cm^−1^ due to casein content in milk [40]. The sugar level was indicated from the bands at 1159–1076 cm^−1^ probably associated with lactose [41]. Milk spectra showed several other peaks between 1200 and 400 cm^−1^ corresponding to other functional groups such as –CH bending, –CO stretching, and –COH in-plane bending due to other milk components like carbohydrates, lipids, amino acids, and organic acids [38].

**Fig. 4:**
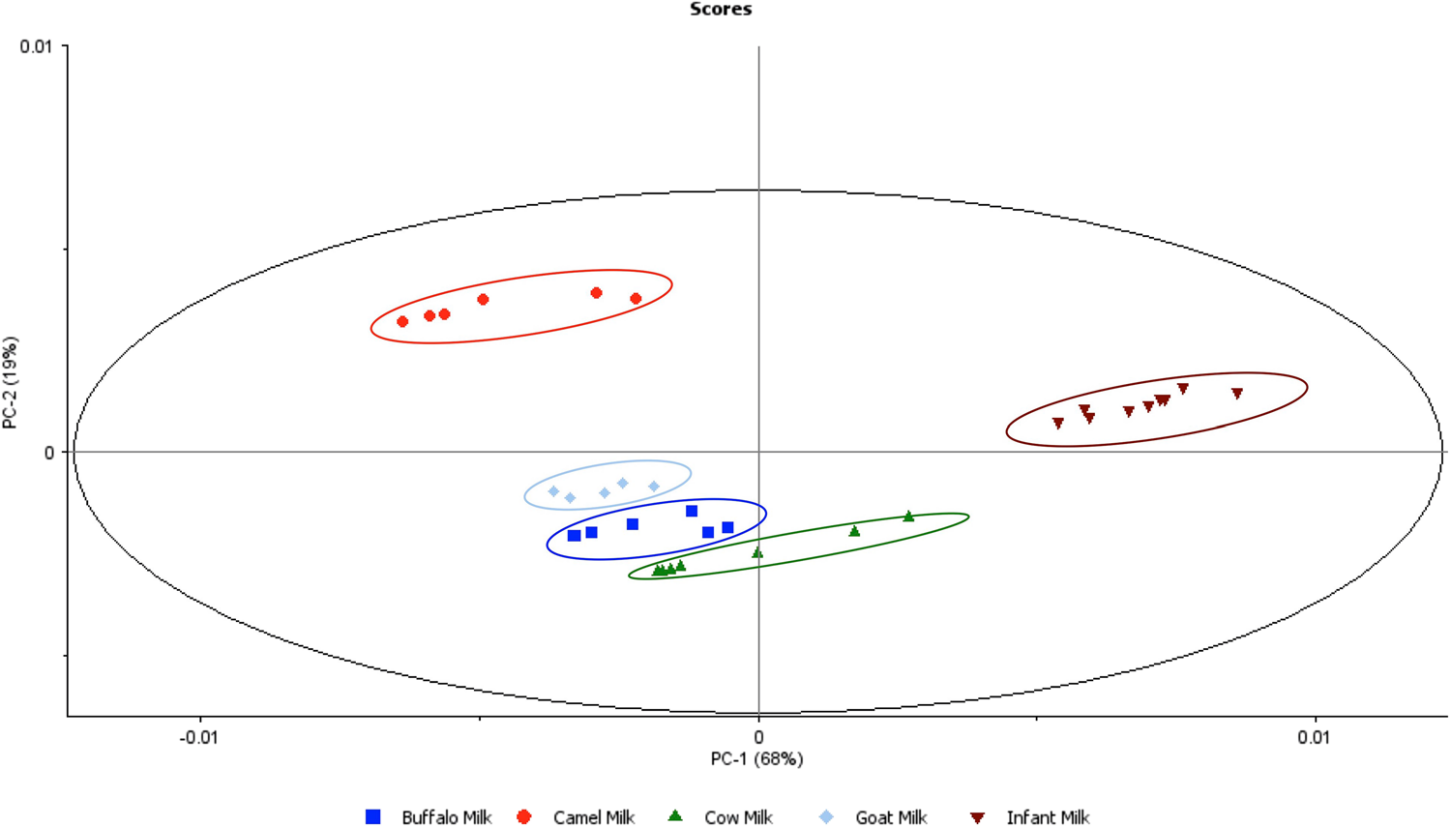
Score plot classification of the five milk types studied according to principal component analysis

Furthermore, using principal component statistical analysis (PCA), the measured SEIRA spectra were able to classify milk samples, resulting in five distinct groups as shown in Fig. 4.

Any contamination or impurities in milk can be easily discovered as the impurities would lead to the appearance of abnormal bands on the unique IR spectrum of raw milk samples; i.e., the unique fingerprint of each milk will be altered. Urea is a natural metabolic constituent of raw milk, and it has a maximum limit imposed by the Food Safety and Standards Authority of India (FSSAI) Act 2006 in the Prevention of Food Adulteration (PFA) Rules 1955, which is 70 mg per 100 mL. Commercial urea can be added to raw milk as an adulterant to increase solid nonprotein nitrogen content [42]. The developed SEIRA-ANN method can be used for the quantitative determination of the level of urea in raw milk.

#### The effect of localized surface plasmon resonance on FTIR bands

For qualitative discrimination between milk types and visual comparison of the levels of its main components, milk solutions were placed separately over the ATR unit crystal and scanned to get results after 1 min as presented in Fig. 3. While the milk sample is rich in multiple components and metabolites such as lactose and urea [41], several of these are at low levels, warranting more advanced techniques such as SEIRA for the determination of main milk macronutrients.

**Fig. 5:**
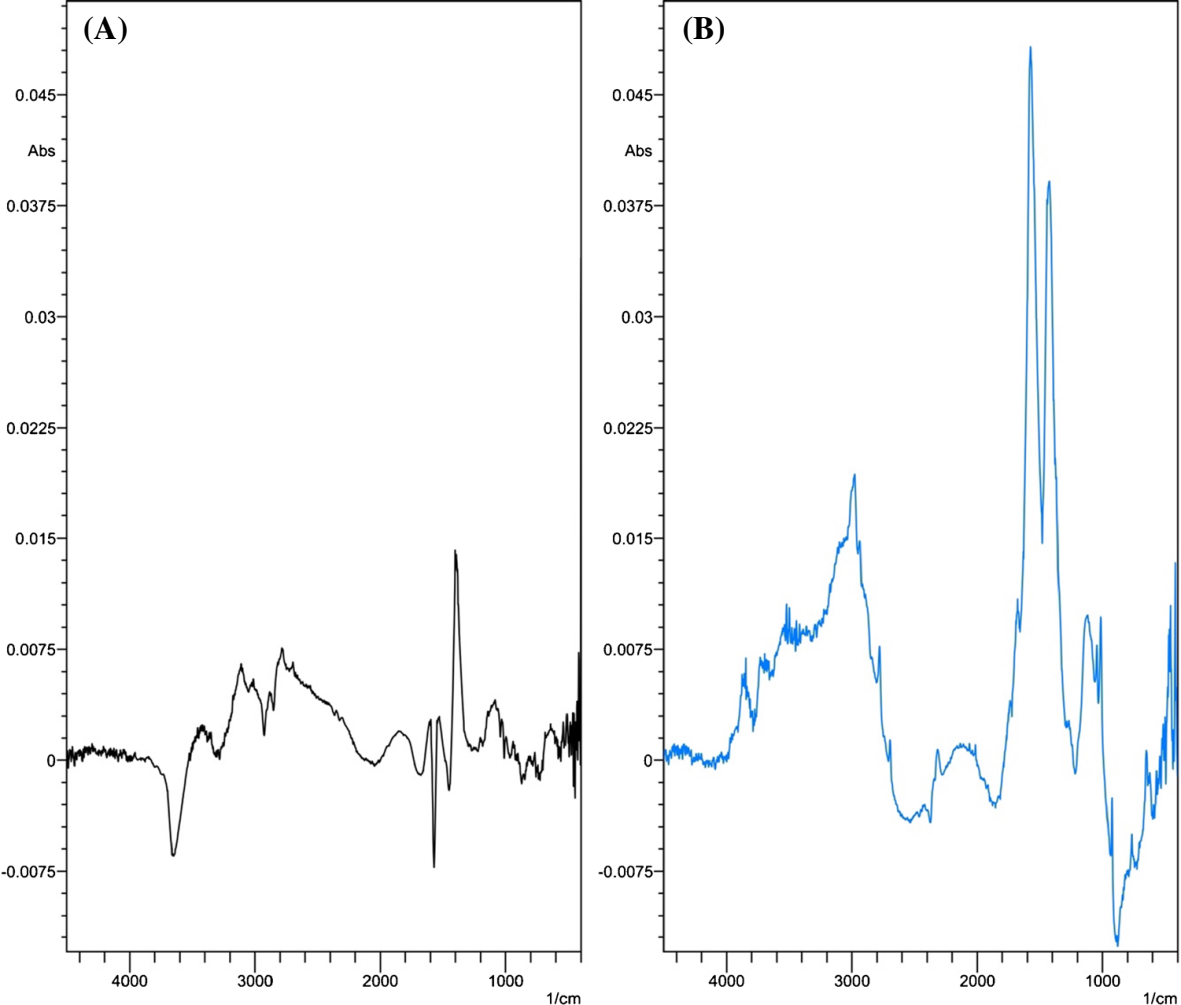
FTIR spectrum of a 300-µL raw buffalo milk sample placed over a glass slide (**A**). SEIRA spectrum of a 300-µL raw buffalo milk sample placed over a glass slide coated with citrate-capped silver nanoparticles (**B**)

SEIRA represents a variation from the conventional infrared spectroscopy exploiting the signal enhancement generated by the LSPR of the thin films of Cit-AgNPs. When infrared light passes through the ZnSe ATR prism, it is reflected nearly 12 times, producing evanescent IR waves which face Cit-AgNP islands fixed on the glass substrate. These waves interact with the Cit-AgNPs and their surrounding milk components, leading to a LSPR enhancement accompanied by the magnification of FTIR signals. The deposited Cit-AgNPs on the glass slides formed several nanoparticle islands that provided the LSPR amplification of the infrared absorption bands. A simple experiment was performed for optimization of the nanoparticle film thickness by soaking five clean salinized glass slides for durations of 1, 2, 4, 10, and 12 h into a beaker containing a freshly prepared solution of Cit-AgNPs. When the suitable duration passes, the immersed slides were removed slowly, and they were placed in an oven at 100 °C for 10 min. SEM micrographs were recorded on the surface of the five slides to identify the shape and size of the nanoparticles on the surface of the glass slides. It was found that a uniform thin film attached to the surface of the slides was obtained after 12 h of soaking. The SEM micrograph in Fig. 2 shows Cit-AgNPs which are uniform in shape, spherical/semispherical with a smooth surface, and no irregular shapes related to any impurities were observed. These results were consistent with the results obtained while examining the magnitude of FTIR peak enhancement of the buffalo milk samples. The spectra of buffalo milk samples were recorded on the surface of Cit-AgNP-coated glass slides without nanoparticles and other glass slides without nanoparticles. Figure 5 reveals that soaking of the salinized glass slides into a solution of citrate-capped silver nanoparticles for 12 h was enough to form metal islands that can enhance the FTIR signals of milk samples by threefold and in accordance with SEM micrographs of the soaked slides (Fig. 2).

SEIRA and SERS are based on the interaction between the nanoparticle’s surface and the under-investigation molecules. Several mechanisms have been proposed for describing the interaction between the nanoparticle’s surface and the molecules that can bind to it. The mechanisms depend on hydrophobic interactions, chemisorption processes, and electrostatic attractions. The identification of the suitable mechanism of interaction is not straightforward, but without the localized surface plasmon electromagnetic enhancement, there would be no signal, but the interaction mechanism determines what is observed as stated by Otto [43]. This confirms the fact that the obtained SEIRA spectra include information about the under-investigation molecules and the surrounding environment, in particular the nature of the interactions with the substrate nanoparticles [44]. The effect of the LSPR of the Cit-AgNPs was not limited to FTIR signal magnification, but also to some peak shifts in the presence of the nanoparticles that do not exceed ± 3 cm^−1^ in comparison to the FTIR spectrum recorded without Cit-AgNPs (Fig. 5). The shifts indicate hydrophobic interactions, chemisorption effects, and electrostatic attraction with Cit-AgNPs [45]. The functional groups which exist in the chemical structure of the investigated milk components likely to be involved in surface interactions with Cit-AgNPs are –C = O, –COOH, and –NH). These interactions allowed for more stabilizations of the components on the surface of the negatively charged nanoparticles.

### ANN model predictive ability

ANN is one of the most important prognostic chemometric methods used to find solutions when other statistical methods are not applicable. It has several advantages such as the ability to learn from examples, real-time analysis, and accurately fitting nonlinear calibration. Despite the fact that ANN with the proper topology can model linear calibration problems, it is well known and strongly recommended for nonlinear calibration problems when the prediction samples are within the calibration domain. On other hand, it is not recommended to use ANN as the first option for calibration purposes due to higher susceptibility to overfitting. Overfitting occurs when a model tries to predict a trend in data that is too noisy. So that we have tried a linear regression model such as partial least squares (PLS) before applying the ANN model. The tried PLS models were evaluated during method optimization using several parameters such as *RMSEC*, *RMSECV*, *RMSEP*, and coefficient of cross-validation. Based on the obtained PLS modeling results, the model was acceptable for the determination of most of the under-investigation milk components (fat, casein, and lactose) while urea was only acceptable for qualitative screening purposes. The PLS regression analysis results obtained for urea suggest that the relationship between the extinction FTIR peaks and urea concentration had a sigmoidal nonlinear shape which may be due to the relatively low concentrations of urea and its nature as a metabolite that is affected by the animal feeding. In this sense, ANN modeling was developed for the quantitative determination of milk components (fat, casein, lactose, and urea) as it is suitable for handling nonlinear data and improving the accuracy and predictability of the model. To prevent overfitting while training the ANN model, we have ensured simplification of the experimental model, early stopping of training, data regularization, augmentation, and dropouts.

**Fig. 6:**
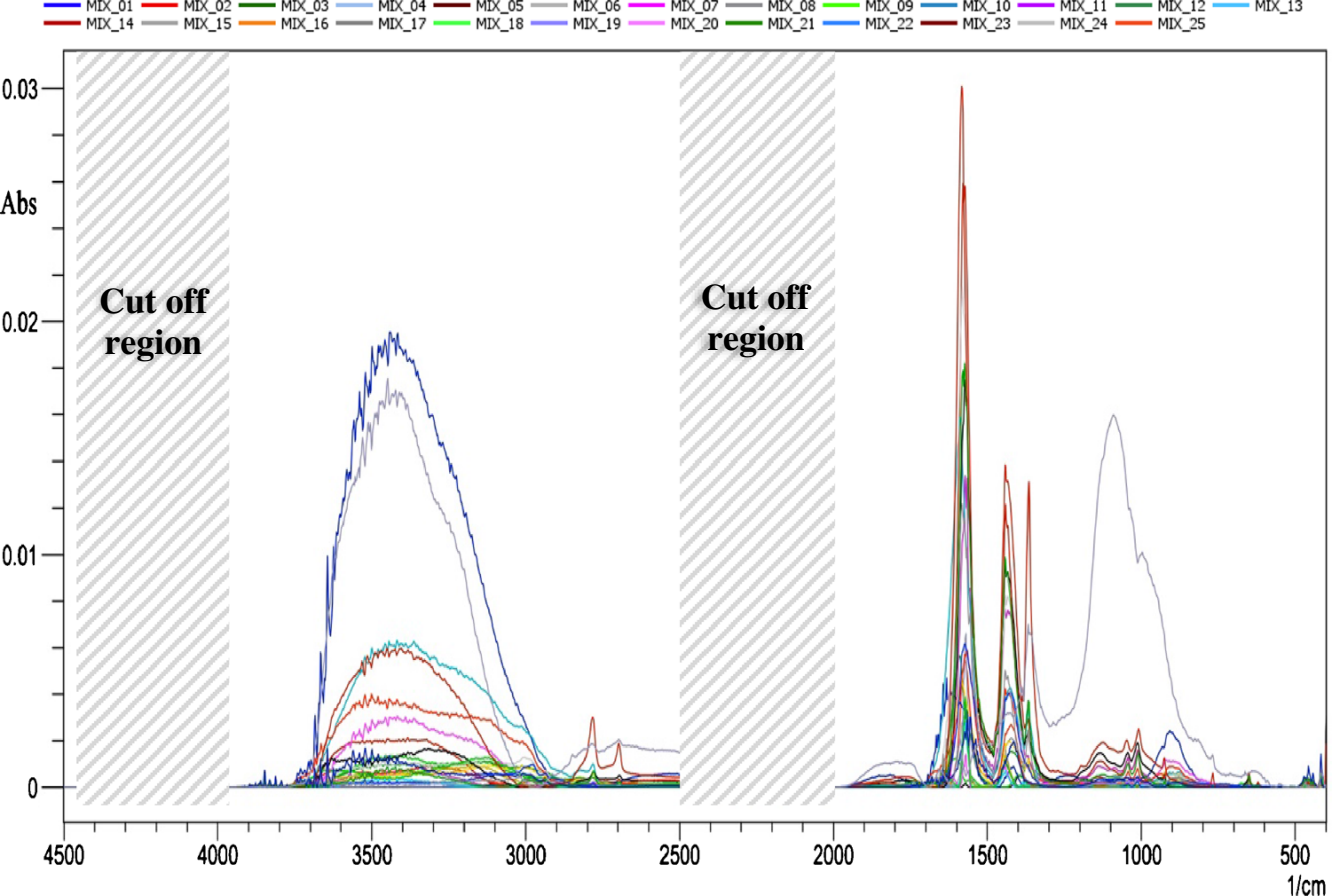
SEIRA spectra of the 25 mixtures which were used for building the ANN model. The mixture concentrations are described in Supp. Table 1S

The simultaneous quantitative determination of the main milk components such as fat, casein, lactose, and urea was a challenge, especially because these components are present at a wide concentration levels in such a matrix of milk. The approximate composition of a typical milk sample was reported in the literature [3, 29, 30], containing fat (1.5–7.5% w/w), protein (2–6% w/w), casein (1.7–3.5% w/w), lactose (3.8–5.3% w/w), urea (100–400 mg/L), vitamins, some organic acids (0.12–0.21% w/w), and water (85.3–88.7% w/w). These values were taken into consideration during the experimental design of the calibration, validation, and test sets to get a representative ANN model with the best predictive ability. The multilevel partial factorial design has been selected for building the experimental set as it offers many of the advantages of the complete factorial design while requiring a considerably smaller number of experiments. Only 25 mixtures consisting of uncorrelated five concentration levels for each one of the four milk components described in Suppl. Table 1S were enough for the experimental design. Table 1 shows the selected concentration ranges of 1.5–7.5%, 1–5%, 1.5–7.5%, and 100–400 mg/L for fat, casein, lactose, and urea, respectively. The mixtures were successfully placed over the nanoparticle-coated glass slides, and the SEIRA spectra recorded in Fig. 6 illustrate an overlay plot of the 25 prepared mixtures.

**Table 1:** Summary of the statistical values for simultaneous determination of the selected milk components using the optimized ANN model

Parameters	Fat	Casein	Lactose	Urea
Linearity range	1.5–7.5%	1–5%	1.5–7.5%	100–500 mg/L
Intercept	0.00023	0.033	0.047	0.016
Correlation coefficient (*R*^2^)	0.9996	0.9998	0.9989	0.9979
Root mean square error of calibration	0.33	0.45	0.22	0.32
Root mean square error of prediction	0.29	0.41	0.25	0.33

A pre-processing step of the obtained spectra is considered a critical step as the failure manipulation of the data may generate noise and/or damage the important informative peaks. The spectrum of each component (fat, casein, lactose, and urea) was recorded and compared to the original milk spectra to find the informative regions as shown in Suppl. Figure 4S. Different pre-processing modes were examined such as derivatization, normalization, baseline correction, and de-trending. The baseline correction was the best pre-processing accompanied by cutoff and discarding of the IR absorption bands between 4500–4000 and 2500–1900 cm^−1^ as these contain noise due to humidity and CO_2_ interference. These manipulated spectra were used to build the ANN model.

The applied type of ANN model was a feed-forward network trained with the backpropagation is a scientific term used to describe ANN model. The recorded SEIRA spectra of the mixtures were used as the input layer and the 25 concentration levels as the output layer during the building of the ANN model. The input layer was composed of an array of size *P*_*ij*_ (2128 × 25) belonging to the set of SEIRA spectra of the mixtures, where its rows represent the absorbance for each of the 2128 wave numbers acquired in each spectrum. The ANN model training was performed by random selection of 70% of the experimental set as a training calibration set, 10% as a test set, and 20% as internal cross-validation data set. The random selection was repeated several times until the optimum ANN model was obtained as described in the flowchart in Fig. 1. Then, an external validation set has been used later to validate the selected model. Suppl. Table 2S summarizes the parameters of the proposed ANN model.

There were no simple rules for the selection of the best training algorithm to be used for building the ANN model. Some showed a better result in pattern recognition, and others were faster for solving regression problems. The speed of the algorithms depends on the amount of training data sets, the complexity of network structure, and the problem introduced by the data sets [46, 47]. In the search for the optimum ANN algorithm, different training algorithms were attempted such as Bayesian regularization, Levenberg–Marquardt, and scaled conjugate gradient. Upon loading the experimental set into the ANN toolbox, the same network structure illustrated in Supp. Figure 1S was used with different training functions for each algorithm. Each algorithm was trained several times as illustrated in the workflow presented in Fig. 1 before the selection of the optimum one as several factors have to be optimized such as the number of neurons, hidden layer number, transfer function, and learning coefficient increase and decrease [48]. The determination of the optimum number of neurons in the ANN was the most important parameter in the process of choosing the best network structure. If the number of neurons was too small, the generated output would not cover well the desired targets. If the number of neurons was too high, the network will predict well within the concentration range of the training set but will not be good with unknown concentrations. So, the optimum number of neurons to build the ANN structure was the one that gave the best results with the unknown and known data depending on the outputs of the ANN fed. The ANN network architecture has one hidden layer with 20 neurons and 4 output layers as shown in Supp. Table 2S. The same for the selection of the ANN transfer function as different types of transfer functions have been tested and the purelin–purelin transfer function was selected as it yielded the best results between the input and hidden layers and between the hidden and the output layer. The selected algorithm calculates the error between the actual and the predicted output levels, with such error used for the selection of the optimum ANN model as the model with the lowest error was selected as the calibration model. The Bayesian regularization algorithm typically takes more training time, but it led to good generalization for small or noisy data sets.

The relationship between FTIR extinction bands and the milk component level is a linear relationship with a mean recovery percentile in the range of 98–102%. The model was double validated, with the validation set incorporated in the experimental set and the ANN modeling to stop when the *MSE* of the calibration set decreased concurrently with the increase in the *MSC* of the validation set. Also, an external validation set consisting of 10 mixtures was examined using the ANN model and the obtained mean recoveries were satisfied between 96 and 102%. Table 1 summarizes the statistical values for simultaneous determination of the selected milk components using the optimized ANN model.

### SEIRA-ANN model application to the under-investigation milk samples

Milk analysis was performed with the selected spectral ranges between 400–1900 and 4000–2500 cm^−1^ in the SEIRA region; ANN was able to correctly classify all the under-investigation molecules across all the mid-infrared wave numbers with an error of tolerance equal to ± 2%. As shown in Table 2, the results of the method application are consistent with the reported values in the literature [3, 29, 30, 49], confirming the method’s applicability as an alternative to traditional methods using MilkoScan™. For more validation purposes, the milk samples were analyzed using the MilkoScan™ device following the standard procedure. About 30 mL of milk samples was heated and homogenized for 10 min. Then, the device sampling tubes were immersed in the milk sample to be measured. About 1 min is required to get the results followed by washing steps for about 5 min between samples. Cow, buffalo, goat, camel, and infant formula powdered milk samples were measured separately, and the obtained results were in high agreement with the obtained results using the proposed SEIRA method. Upon statistical comparison, the obtained *t*-test and *F*-test values were less than the reference tabulated values as shown in Table 3.

**Table 2:** Chemical composition of buffalo, cow, goat, camel, and infant’s formula powdered milk using the optimized SEIRA-ANN model

Type of milk	Fat % ± *SD*	Casein % ± *SD*	Lactose % ± *SD*	Urea mg/L ± *SD*
Buffalo	7.60 ± 0.56	4.83 ± 0.76	4.40 ± 0.62	310 ± 0.45
Cow	3.51 ± 0.87	3.68 ± 0.54	4.56 ± 0.66	226 ± 0.19
Goat	5.23 ± 1.03	3.48 ± 0.44	4.16 ± 0.43	350 ± 0.88
Camel	4.81 ± 0.93	3.52 ± 0.27	5.23 ± 0.21	228 ± 0.85
Infants’ powdered	2.40 ± 0.89	1.21 ± 1.12	5.3 ± 0.33	102 ± 0.89

*SD* standard deviation average of three determinations (*n* = 3)

**Table 3:** Statistical comparison of the results of determination of fat, casein, lactose, and urea in different milk types using the optimized SEIRA-ANN model and the traditional MilkoScan™ analyzer

Milk type	Milk composition	SEIRA-ANN	MilkoScan™	*t*-test^b^ (2.306)	*F*-test^b^ (6.39)
		(recover % ± *SD*)	(recover % ± *SD*)^a^		
Buffalo milk	Fat	99.88 ± 0.99	99.23 ± 0.85	1.111	2.180
	Casein	98.97 ± 0.63	100.02 ± 0.87	1.352	1.914
	Lactose	98.45 ± 0.54	99.11 ± 0.61	1.799	1.256
	Urea	101.09 ± 1.12	99.68 ± 1.66	1.263	2.184
Cow milk	Fat	98.98 ± 0.88	98.33 ± 0.81	1.208	1.196
	Casein	98.22 ± 1.20	99.83 ± 1.33	2.006	1.232
	Lactose	100.45 ± 1.61	99.32 ± 0.68	1.441	0.183
	Urea	97.99 ± 1.80	98.55 ± 1.17	0.582	2.333
Goat milk	Fat	98.02 ± 1.09	99.65 ± 1.20	2.244	1.205
	Casein	97.98 ± 1.41	100.01 ± 1.99	1.856	2.005
	Lactose	98.28 ± 0.66	98.21 ± 0.88	0.141	1.809
	Urea	98.67 ± 1.21	99.87 ± 0.71	1.912	2.896
Camel milk	Fat	101.01 ± 0.67	99.97 ± 0.98	1.943	2.215
	Casein	99.66 ± 1.29	98.98 ± 0.89	0.967	2.064
	Lactose	99.89 ± 0.99	98.59 ± 0.79	2.284	1.538
	Urea	98.86 ± 1.10	99.33 ± 0.91	0.734	1.457
Infant powder	Fat	98.32 ± 1.66	100.13 ± 0.96	2.106	2.935
	Casein	98.77 ± 0.66	99.67 ± 0.89	1.803	1.844
	Lactose	98.99 ± 1.71	99.92 ± 0.95	1.062	3.202
	Urea	99.11 ± 1.09	98.89 ± 1.78	0.235	2.670

^a^Average of five determinations

^b^The values in parentheses are the corresponding theoretical values for *t* and *F* at *P* = 0.05

### The SEIRA performance in comparison to MilkoScan™

MilkoScan™ is undoubtedly the most important method in milk analysis. It is widely used for the determination of the main milk components, identification of milk abnormalities, and screening of milk product safety. It can be described as fast, high throughput, accurate, and environmentally friendly. However, MilkoScan™ confronts several difficulties as it requires a minimum of 26 mL of milk for duplicate analysis so, in case of small volumes, dilution steps have to be applied. In addition, these volumes lead to a high amount of waste as a large amount of milk is discarded per day of analysis. Also, cleaning of the MilkoScan™ device tubes and system requires several steps that have to be performed with great caution to prevent contamination of different samples and it requires excessive amounts of cleaning solvents, leading to more waste. The analysis of raw milk using MilkoScan™ needs a manual homogenization step accompanied by heating before each measurement, and the inefficient homogenization may lead to nonuniform distribution of fat globules within the raw milk solution that may lead to light scattering and shift in the frequency of maximum light absorption (known as the Christiansen effect [50]).

In an attempt to mitigate such constraints, we have introduced SEIRA as an alternative to this traditional technique. Once the nanoparticle-coated substrate is prepared, only one drop of milk is sufficient for analysis so minimal sample size is used, and no milk sample pre-treatment is required, leading to less waste generated. Also, the substrates are cheap so they can be discarded or washed with water for reuse, so it is more cost-efficient. The presence of a portable FTIR device permits portable on-site measurements to get results using on-site fresh samples. Both methods can be used for the determination of milk types, so they can be used for discrimination between cow, buffalo, goat, camel, and infant formula powdered milk as the FTIR spectrum is considered a fingerprint for each type of milk.

In SEIRA studies, it is important to bring the under-investigation molecular structures in direct contact or very close to the surface of metallic nanomaterials. For analysis of molecular structures in the milk matrix, colloidal silver and gold nanoparticles were preferred [51–55]. Silver and gold nanoparticles are commonly used nanomaterials for the preparation of SEIRA and SERS substrates, but gold nanoparticles are more expensive than silver nanoparticles and they produce weaker LSPR enhancement [56–60]. Moreover, silver nanoparticles have a higher molar extension coefficient, form metal islands that have a strong SEIRA and SERS effect, have a simple one-step synthesis procedure, and have excellent optical properties [56, 61–63] so we employed silver nanoparticles in the proposed method.

By comparing the performance of the developed SEIRA-ANN method with already published nanomaterial-based methods, it can be concluded that the developed method has a good performance for the determination of analytes in the milk matrix as illustrated in Table 4. Moreover, the coupling with artificial neural networks facilitates the simultaneous determination of the main milk compounds in each of the five milk types, adding a new quality control test and identification of milk adulteration. The obtained SEIRA spectra can be used for the detection of milk abnormalities or adulteration by detecting any alteration in the characteristic FTIR spectrum due to the presence of adulterants, impurities, or interfering substances in the milk sample. In future work, we will consider more techniques such as array-based sensing tactics as it combines the response from the interactions between sensors and analytes to generate a distinct pattern (fingerprint) for each analyte.

**Table 4:** Nanomaterial-based methods for determination of analytes in different milk types in comparison to the current method

The used nanomaterial	Technique	The analyte	*LOD*		Linear range	Ref
Magnetic nanoparticles with immobilized captured antibodies	Potentiometry	*Salmonella typhimurium* in milk samples	1100 cells per mL		10 to 10^8^ cells per mL	[64]
Poly A aptamer and silver nanoparticles	Colorimetric bioassay	Tobramycin in milk	70 pM		0.1–100 nM	[51]
Metalloporphyrin and gold nanoparticles modified hollow zeolite imidazole Framework-8	Colorimetric assay	Choline in infant formula milk powder	0.05 mM		0.05–2.0 mM	[52]
Amorphous carbon nanoparticles	Lateral flow assay strips (immunoassay)	Adulteration of cow’s milk with buffalo’s milk	Below 1% adulteration		1 to 100%	[65]
Covalent organic framework capped with sliver nanoparticles	SERS	Benzoic acid in liquid milk	0.13 μg/mL		2–20 μg/mL	[53]
β-Cyclodextrin-functionalized silver nanoparticles	SERS	Norfloxacin in milk	1.701 ng/mL		7.98–159.67 ng/mL	[54]
Lanthanide-functionalized metal–organic frameworks	Fluorescence	Antibiotics in milk	19.159 ng/mL		127.7–6386.6 ng/mL	[66]
Citrate-stabilized gold nanoparticle	UV/Vis spectrometer	Melamine in milk	0.05 mg/L		0.1–2 mg/L	[55]
Citrate-capped silver nanoparticles	SEIRA-ANN	Fat, casein, urea, and lactose in milk samples of cow, camel, goat, buffalo, and infant formula	Fat	0.74%	1.5–7.5%	*Current work*
			Casein	0.52%	1–5%	
			Lactose	0.35%	1.5–7.5%	
			Urea	21 mg/L	100–500 mg/L	

## Conclusion

In this study, the potential of two measurement modes of vibrational spectroscopy was evaluated and compared for the qualitative and quantitative analysis of different milk types and components. The performance of FTIR analysis using the traditional MilkoScan™ device was compared to the performance of the newly developed SEIRA-ANN nanoparticle-based method. Both techniques showed a good performance for the quantitative determination of the main milk components (fat, casein, urea, and lactose) of the examined cow, camel, goat, buffalo, and infants’ formula milk samples. SEIRA coupled to the ANN chemometric tool has more advantages as the coupling to chemometric techniques such as ANN permits quantitative analysis without any prior sample pre-treatment and any destructive sample manipulation. Such ANN treatment of spectral data was faster, simpler, and more convenient than the usual technique. The prediction and application results of the prepared SEIRA silver nanoparticle–coated glass substrates provided good estimation of the milk components as reflected in the low errors of predictions and good recoveries in addition to suitability to distinguish between milk types and the identification of adulteration. The main limitation that may face this technique is that it requires intimate contact between the enhancing surface and the sample placed on the substrate; this problem can be fixed by using a pressure clamp during the measurements. Also, the prepared nanoparticle-coated substrates may degrade with time, leading to a decrease in the enhancement signal and limiting the reusability of the substrate. Despite its limitations, the sensitivity of SEIRA, as well as its exceptional spectral selectivity, has made SEIRA an attractive technique to detect a wide range of chemical species.

## Supplementary Information

Below is the link to the electronic supplementary material.

Supplementary file1 (DOCX 1333 KB)
