# Supplementary material for: Identification of milk quality and adulteration by surface-enhanced infrared absorption spectroscopy coupled to artificial neural networks using citrate-capped silver nanoislands
Source: Mikrochim Acta. 2022 Jul 29;189(8):301. doi: 10.1007/s00604-022-05393-4 (PMC9338147; doi:10.1007/s00604-022-05393-4)
Supplement: Supplementary file 1 — Supplementary file1 (DOCX 1333 KB) [file 604_2022_5393_MOESM1_ESM.docx]

**Electronic Supplementary Material**

**Identification of milk quality and adulteration by Surface-enhanced infrared absorption spectroscopy coupled to artificial neural networks using citrate-capped silver nanoislands**

**Sherif M. Eid ^a^*, Sherin el Shamy ^b^, Mohamed A. Farag ^c,d^**

*^a^ Analytical chemistry department, Faculty of Pharmacy, October 6 University, 6 October City, Giza, Egypt.*

*^b^ Pharmacognosy Department, Faculty of Pharmacy, Modern University for Technology & Information, Cairo, Egypt*

*^c^ Pharmacognosy Department, Faculty of Pharmacy, Cairo University, Cairo, 11562, Egypt*

*^d^ Chemistry Department, School of Sciences & Engineering, The American University in Cairo, New Cairo 11835, Egypt*

*Corresponding Author, Email: [Sheriefmohammed@o6u.edu.egm](mailto:Sheriefmohammed@o6u.edu.egm), [sherief055@icloud.com](mailto:sherief055@icloud.com)

**Preparation method of Citrate capped silver nanoparticles**

A slightly modified the Lee-Meisel method [1] has been used for the preparation of Cit-AgNPs colloidal solution. In a 500-ml clean beaker, 36 mg of AgNO_3_ was completely dissolved in 350 ml of double distilled water. The solution was heated and kept at kept at 65 ºC. Another solution of tri-sodium citrate (5%) was freshly prepared, then 5 ml were added to the hot solution with continuous stirring. The formation of Cit-AgNPs can be observed by the appearance of yellow colored solution.


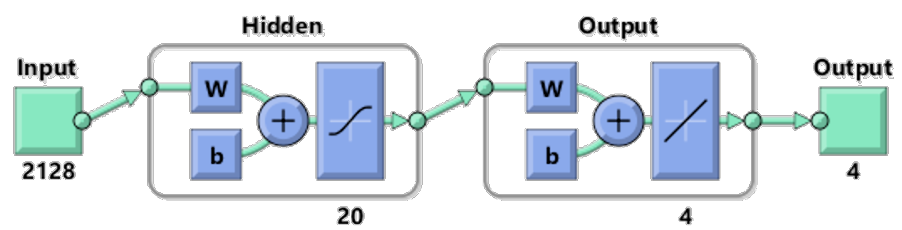


**Fig. 1S:** The simplified presentation of the ANN structure used in all our algorithms for the determination of concentration of milk components.


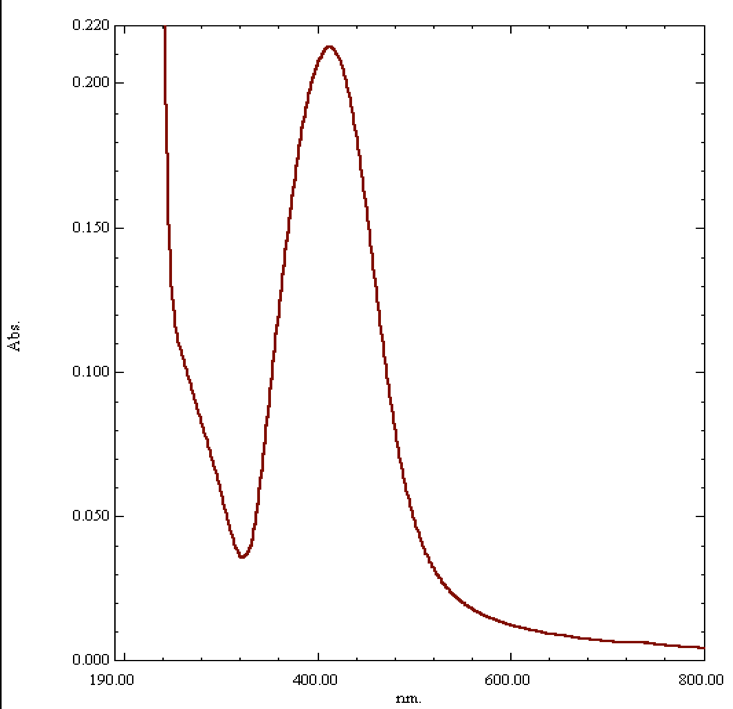


**Fig. 2S:** The Localized surface plasmon resonance extinction UV band of the prepared citrate capped silver nanoparticles showing a λ_max_ at 411 n m.


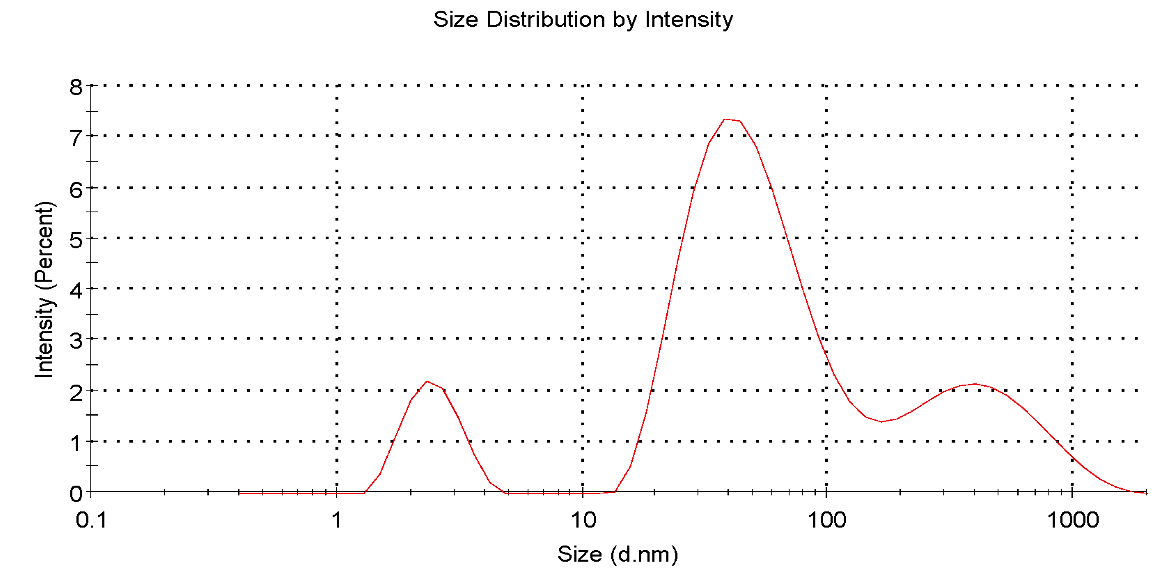


**Fig. 3S:** Dynamic light scattering of the prepared citrate capped silver nanoparticles size distribution by intensity.


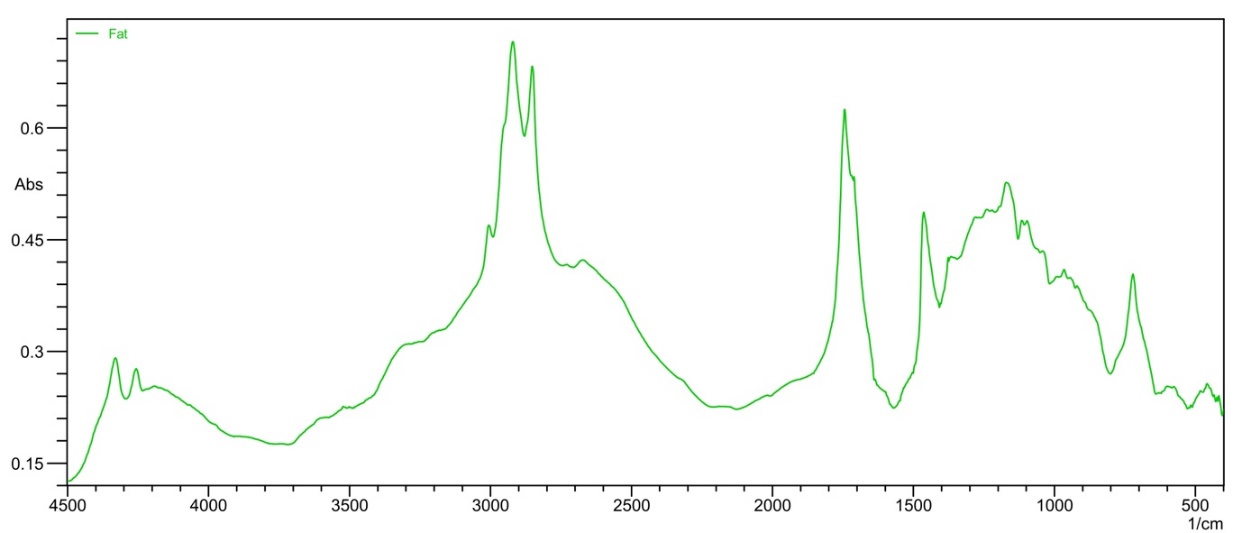


A)


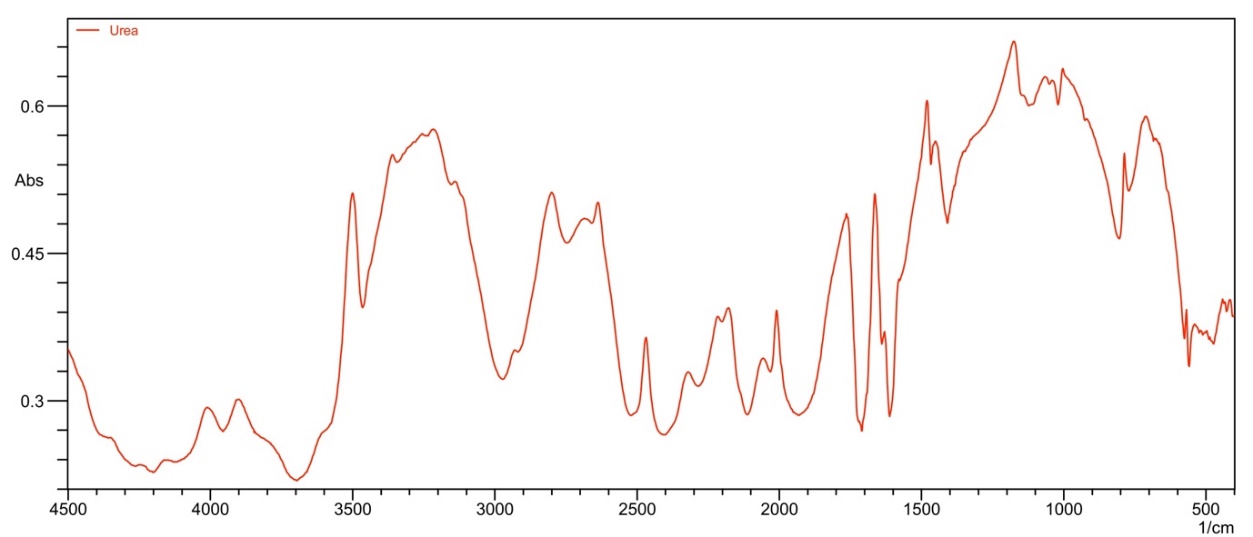


B)


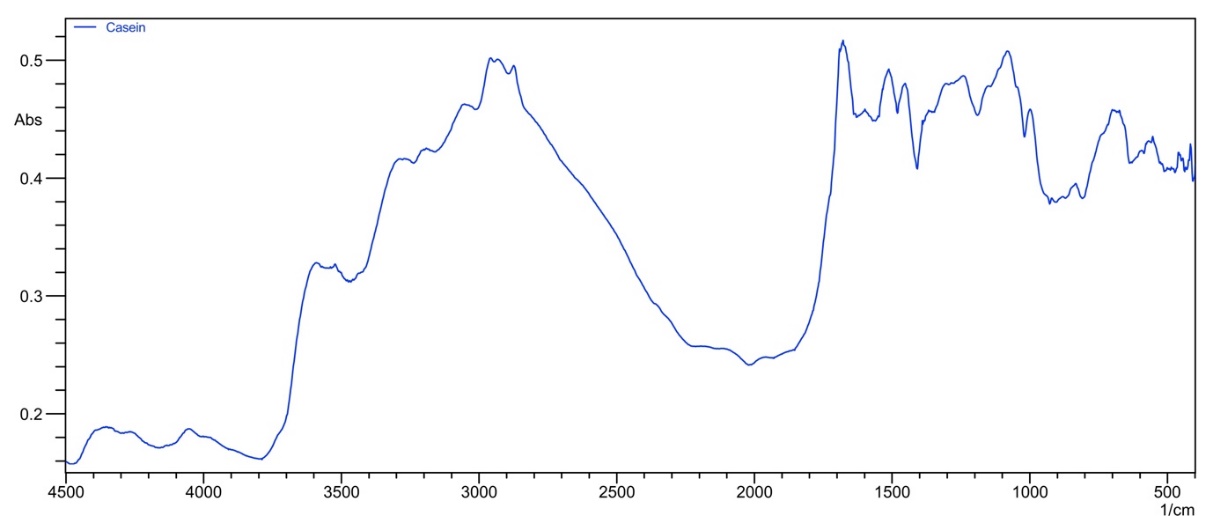


C)


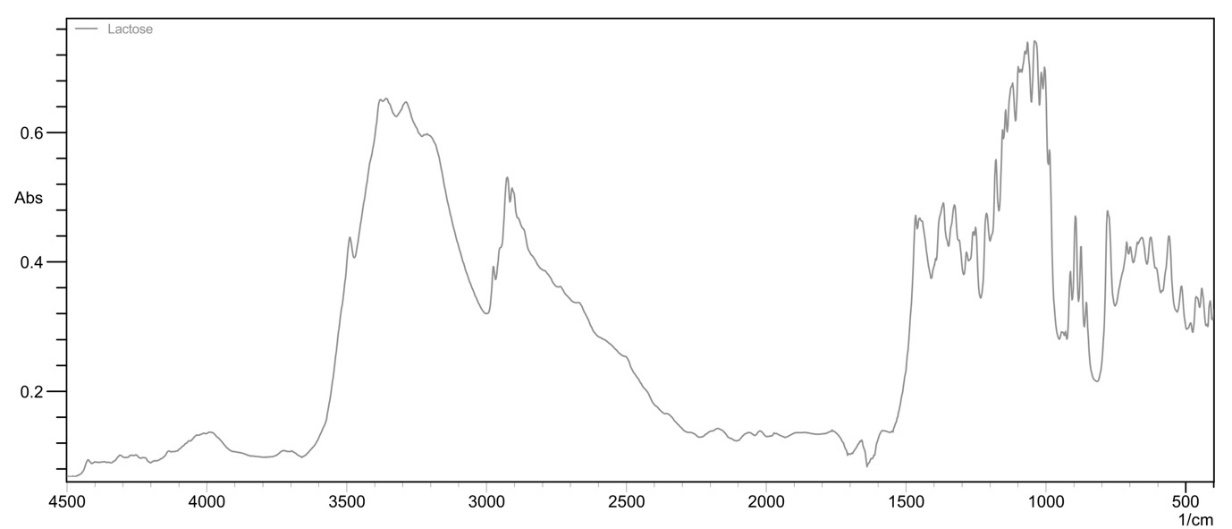


D)


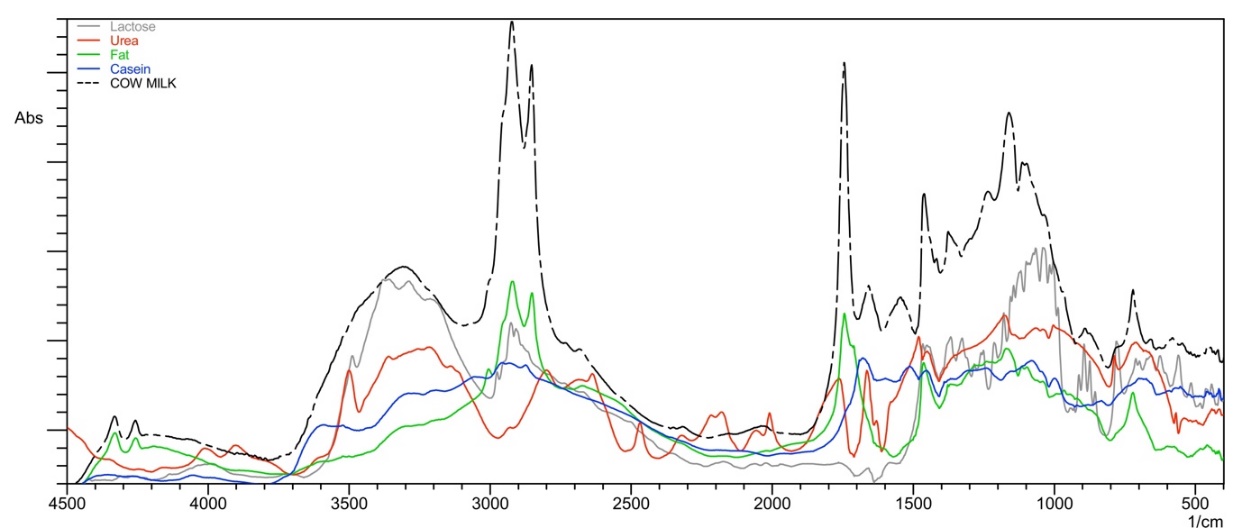


E)

**Fig. 4S** FTIR Extinction spectra (SEIRA) of the different milk components when placed over the nanoparticles coated glass substrate: A) fat, B) urea, C) casein, D) lactose, and E) overlay plot of the four milk components and one of the milk sample (cow milk).

**Table 1S:** The coded levels and concentration ranges of the selected milk components (fat, casein, lactose, and urea).

| ***Code Levels*** | | | | ***Mixture***  ***numbers*** | ***Concentration (mg/ml)*** | | | |
| --- | --- | --- | --- | --- | --- | --- | --- | --- |
| Fat | Casein | Lactose | Urea |  | Fat | Casein | Lactose | Urea |
| 0 | 0 | 0 | 0 | **1** | 45 | 30 | 45 | 0.3 |
| 0 | −2 | -1 | -2 | **2^*^** | 45 | 10 | 30 | 0.1 |
| −2 | −2 | -2 | 2 | **3** | 15 | 10 | 45 | 0.5 |
| −2 | 2 | 2 | 2 | **4^*^** | 15 | 50 | 75 | 0.5 |
| 2 | −1 | 2 | 0 | **5** | 75 | 20 | 75 | 0.3 |
| −1 | 2 | 0 | -1 | **6^*^** | 30 | 50 | 45 | 0.2 |
| 2 | 0 | -1 | 2 | **7** | 75 | 30 | 30 | 0.5 |
| 0 | −1 | 2 | -1 | **8^*^** | 45 | 20 | 75 | 0.2 |
| −1 | −1 | -1 | 1 | **9** | 30 | 20 | 30 | 0.4 |
| −1 | 1 | 1 | 1 | **10^*^** | 30 | 40 | 60 | 0.4 |
| 1 | 2 | 1 | 0 | **11** | 60 | 50 | 60 | 0.3 |
| 2 | 1 | 0 | 2 | **12** | 75 | 50 | 45 | 0.5 |
| 1 | 0 | 2 | 1 | **13^*^** | 60 | 30 | 75 | 0.4 |
| 0 | 2 | 1 | 2 | **14** | 45 | 50 | 60 | 0.5 |
| 2 | 2 | 2 | -2 | **15^*^** | 75 | 50 | 75 | 0.1 |
| 2 | −2 | -2 | -2 | **16** | 75 | 10 | 15 | 0.1 |
| −2 | 1 | -2 | 0 | **17^*^** | 15 | 40 | 15 | 0.3 |
| 1 | −2 | 0 | 1 | **18** | 60 | 10 | 45 | 0.4 |
| −2 | 0 | 1 | -2 | **19^*^** | 15 | 30 | 60 | 0.1 |
| 0 | 1 | -2 | 1 | **20** | 45 | 40 | 15 | 0.4 |
| 1 | 1 | 1 | -1 | **21^*^** | 60 | 40 | 60 | 0.2 |
| 1 | −1 | -1 | -1 | **22** | 60 | 20 | 30 | 0.2 |
| −1 | −2 | -1 | 0 | **23** | 30 | 10 | 30 | 0.3 |
| −2 | −1 | 0 | -2 | **24** | 15 | 20 | 45 | 0.1 |
| −1 | 0 | -2 | -1 | **25** | 30 | 30 | 15 | 0.2 |

#### ^*^ The validation set concentrations.

**Table 2S.** Optimized parameters of ANNs in the proposed chemometric model

| **Parameters** | **Values** |
| --- | --- |
| **Architecture** | 17-5-3 |
| **Training algorithm** | Bayesian regularization |
| **Transfer function** | Purelin–Purelin |
| **Number of hidden layers** | 1 |
| **Number of neurons** | 20 |
| **Learning coefficient** | 0.001 |
| **Learning coefficient decrease** | 0.001 |
| **Learning coefficient increase** | 100 |

***References***

[1] D. Gkogkou, T. Shaykhutdinov, C. Kratz, T.W.H. Oates, P. Hildebrandt, I.M. Weidinger, K.H. Ly, N. Esser, K. Hinrichs, Gradient metal nanoislands as a unified surface enhanced Raman scattering and surface enhanced infrared absorption platform for analytics, The Analyst, 144 (2019) 5271-5276.
